# The Carotenogenesis Pathway via the Isoprenoid-β-carotene Interference Approach in a New Strain of *Dunaliella salina* Isolated from Baja California Mexico

**DOI:** 10.3390/md7010045

**Published:** 2009-02-10

**Authors:** J. Paniagua-Michel, Willian Capa-Robles, Jorge Olmos-Soto, Luis Enrique Gutierrez-Millan

**Affiliations:** 1 Department of Marine Biotechnology, Centro de Investigación Científica y de Educación Superior de Ensenada, Ensenada 22860, México; 2 Present address: Facultad de Ciencias, Universidad Nacional del Santa. Av. Universitaria S/N, Bellamar, Chimbote, Peru

**Keywords:** bioactive compounds

## Abstract

*D. salina* is one of the recognized natural sources to produce *β*-carotene, and an useful model for studying the role of inhibitors and enhancers of carotenogenesis.  However there is little information in *D. salina* regarding whether the isoprenoid substrate can be influenced by stress factors (carotenogenic) or selective inhibitors which in turn may further contribute to elucidate the early steps of carotenogenesis and  biosynthesis of β-carotene. In this study, *Dunaliella salina* (BC02) isolated from La Salina BC Mexico, was subjected to the method of isoprenoids-β-carotene interference in order to promote the interruption or accumulation of the programmed biosynthesis of carotenoids. When Carotenogenic and non-carotenogenic cells of *D. salina* BC02 were grown under photoautotrophic growth conditions in the presence of 200 µM fosmidomycin, carotenogenesis and the synthesis of β-carotene were interrupted after two days in cultured *D. salina* cells. This result is an indirect consequence of the inhibition of the synthesis of isoprenoids and activity of the recombinant DXR enzyme thereby preventing the conversion of 1-deoxy-D-xylulose 5-phosphate (DXP) to 2-C-methyl-D-erythritol (MEP) and consequently interrupts the early steps of carotenogenesis in *D. salina*. The effect at the level of proteins and RNA was not evident. Mevinolin treated *D. salina* cells exhibited carotenogenesis and β-carotene levels very similar to those of control cell cultures indicating that mevinolin not pursued any indirect action in the biosynthesis of isoprenoids  and had no effect at the level of the HMG-CoA reductase, the key enzyme of the Ac/MVA pathway.

## 1. Introduction

Carotenoids, whose bioactive role has become the object of considerable scientific and public attention in the last years, are a rich source of antioxidants used commercially in cosmetics and food supplements for both animals and humans. Recently, the consumer demand for natural β -carotene has increased and epidemiological studies suggest anti-cancer activity of this pro-vitamin A pigment. β-carotene is massively accumulated  (up to 10% on dry weight basis) within globules in the inter-thylakoid spaces of the chloroplast of the halophilic microalgae *Dunaliella salina*  [1]. *D. salina* is considered the best commercial source of natural β-carotene in the world [2]. The molecule of β-carotene is made up of eight isoprene units, which are cyclised at each end. The configuration of each double-bond can occur in different geometrical forms in D. salina [3]: all trans (**1**) and 9-cis isomers (**2**) .

**Figure:**
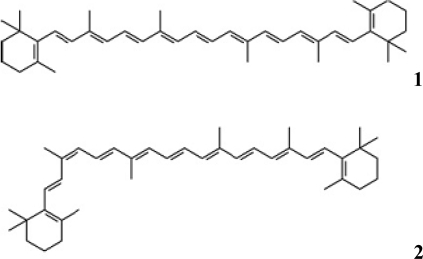

Several studies have shown that in *Dunaliella*, β-carotene functions as a means of storing carbon and quenching oxygen singlets. In addition, β-carotene globules have been implicated in photoprotection or absorption in the periphery of the chloroplast [4].

Preliminary investigations showed that the regulation of carotenoid biosynthesis in *D. salina* in almost all instances was related to deficiency in nitrate, sulfate, and phosphate in the culture media as well as high light intensity and high sodium chloride concentration [5–7].

Isoprenoids are synthesized by condensation of their isopentenyl diphosphate pools (IPP), considered  the universal precursor of five-carbon building block in the biosynthesis of all carotenoids and β-carotene in plants and algae [8]. Two pathways for these precursors are known: the mevalonate pathway occurring in eucaryotes, Archeobacteria and cytosol of higher plants and the recently discovered non-mevalonate pathway (also known as 1-deoxy-D-xylulose 5-phosphate (DXP) pathway or 2-C-methyl-D-erythritol (MEP) [9, 10].

The studies concerning the early steps of carotenogenesis followed by *Dunaliella salina* are scarce and there is less information regarding whether the isoprenoid substrate can be influenced by  carotenogenic conditions or selective inhibitors, *viz*, mevinolin and fosmidomycin. This approach can be useful to improving production level by manipulating precursor enhancers, to control downstream isoprenoid and carotenoids and further advance in the biosynthesis of β-carotene and carotenogenesis in *D. salina*. The present research was aimed at evaluating the isoprenoid-β-carotene interference approach by the action of inhibitors in the programmed synthesis of the early steps of carotenogenesis and β-carotene accumulation in an indigenous strain of *Dunaliella salina* isolated from La Salina, Ensenada, B.C. (Mexico).

## 2. Materials and Methods

### 2.1 Dunaliella salina strain and Carotenogenesis

Cells of *D. salina* isolated from La Salina B.C. (32° 05’; 118° 40’) in the Northwest coast of México were used in this study. The isolated strain was correctly assigned by [11], lately corroborated with molecular biological techniques [12] and maintained under the acronym *Dunaliella salina* BCO2 in the strains collection of the Department of marine Biotechnology. Cells were maintained in a growth médium (control) containing, unless otherwise stated, 1 M NaCl, 5 mM MgSO_4_, 0.3 mM CaCl_2_, 5 mM NaNO_3_, and 0.2 mM KH_2_PO_4_ at pH 7.5–8, and a mixture of micronutrients, as previously described [13]. *D. salina* cells were collected by centrifugation, then they were transferred to carotenogenic media containing 2.5 mM NaNO_3_ and 2 M NaCl respectively. Bacteria free cultures were developed by successive generational growth under continuous illumination with cool-white fluorescent lamps at 382 μE/m^2^/s, and maintained at 20 ± 2 ºC. Three experimental replicate were set-up for each treatment.

### 2.2 Inhibitors of Carotenogenesis

The two carotenogenesis inhibitors were used: mevinolin, an inhibitor of the 3-hydroxy-3-methylglutaryl-coenzyme A reductase in the mevalonate pathway and  fosmidomycin, an inhibitor of 1-deoxy-D-xylulose-5-phosphate reductoisomerase that suppresses the biosynthesis of isoprenoids and accumulation of carotenoids (β-carotene) respectively in the non-mevalonate pathway. Mevinolin was purchased from Sigma (USA), which was previously converted to the water-soluble sodium salt as described in [14]. Fosmidomycin was purchased from Molecular Probes (USA) and was dissolved in culture medium prior to application to the Dunaliella cultures. The active concentration of inhibitors mevinolin (1μM) and fosmidomycin (200 μM) was determined previously in algal cultures at a cell density of 10^6^ cells/ml. Aliquots were taken from the stock solutions in order to obtain the active concentration for each inhibitor, *viz*, control medium added with 1 μM mevinolin. In the case of fosmidomycin we added to the control medium the following concentrations of fosmidomycin: 50, 100, 150 and 200 μM, unless otherwise stated.

### 2.3 Growth and pigments

Cell grown was determined by counting immobilized cells (by Lugol) with a hemocytometer. For total chlorophyll and carotenoids determination, the centrifuged algae obtained (3000 X g) was resuspended in 90% acetone in water and stirred by vortex. After filtration, chlorophyll was  determined by reading at 665 nm the absorption spectra of the extracts recorded in a Hewlett-Packard Diode-Array spectrophotometer. The absorbances of the extract at 450 nm were used assuming that 1 (%) (w/w) of β-carotene have an average extinction coefficient of 2600 cm^–1^. Pigments separation was performed by using a Hewlet Packard (Agilent) series 1100 HPLC (Agilent technologies Palo Alto, CA) with a C18 reverse phase 300SB-C18 Zorbax column (5 µ, 150 mm). An isocratic step was eluted with 85:10:5 acetonitrile, methanol and dichloromethane (v/v/v) respectively at a flow rate of 1 mL/min [15]. Standard of β-carotene and Chlorophyll were ran under the same conditions.

### 2.4. Protein measurements

Protein content of Dunaliella was determined according to [16]. The algae were collected by centrifugation at 3500 X g for 15 minutes at 10ºC. Thereafter the samples pellets were resuspended in 5 ml NaOH 1N and incubated in a water bath at 100 ºC for 10 minutes followed by a second alkaline extraction for a full protein extraction of the cell concentrates [17].  Protein measurement were carried-out reading at 595 nm and the measurements compared with a calibration curve prepared by using bovine serum albumin following procedures of Bio-Rad protein-assay kit.

### 2.5 Cellular RNA

Total RNA in *Dunaliella salina* BC02 was extracted according to the protocol of [18]. The algae was collected by centrifugation at 13000 g during 15 min. Thereafter, the algal pellet was powdered with a mortar and pestle and added 300 μl of RNA lysis solution (Aquapure RNA Bio-Rad 732-6370) followed by vigorous mixing. All manipulations were carried-out at low temperature conditions. The protein-DNA recovered was placed on ice bath (5 mins) and centrifuged at low temperature ,13000 g.  The aqueous (upper) phase was then transferred into a new tube, re-extracted with 300 μl de isopropanol, and centrifuged (13000 g, 15 min, 20°C) and the supernatant was carefully discarded. The pellet was washed with 70% etanol, centrifuged briefly, and the supernatant was discarded. The pellet was then dried at room temperatura and resuspended in 50 μl RNA hidratation solution (Bio-Rad 732-6370). To determine the RNA concentration, the OD_260_ was measured. A solution of RNA whose OD_260_=1 contains approximately 40 μg.ml-1 [19].

## 3. Results and Discussion

**Figure 1:**
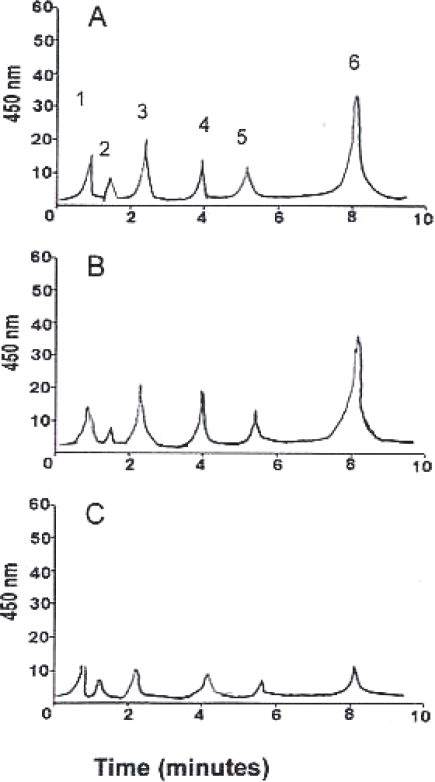
HPLC chromatogram of *Dunaliella salina* cultured in the control growth media (A) after 7 days of culture and in presence of 200 μM fosmidomycin (B) and 1 µM mevinolin (C). The peaks correspond to the following pigments: 1, violaxanthin; 2, neoxanthin; 3, lutein; 4, zeaxanthin; 5, Chlorophyll a; 6, all-trans β-carotene.

**Figure 2:**
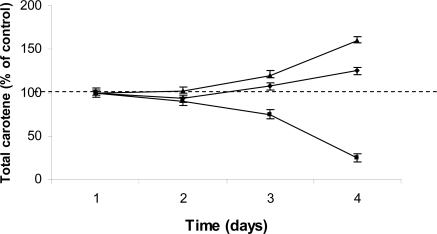
Relative changes in the total carotenoid content during carotenogenesis (line triangle) in *D. salina* expose to fosmidomycin (line square) and mevinolin (line diamond). Data are means ± SD (each point represent n=3).

Preliminary investigations were carried-out in order to determine the optimal concentration of each inhibitor. Application of fosmidomycin, at a range of concentrations from 50 to 200 μM was assessed in cultured *D. salina* BCO2 cells. At concentrations below 150 µM, fosmidomycin had no influence on the biosynthesis of β-carotene or on their number of *D. salina* cells (Table 1), in contrast, when *D. salina* was exposed to 200 μM fosmidomycin caused growth inhibition and arrested the carotenoids accumulation that was apparent at day 7 of cultivation (Figure 1C). Mevinolin, did not exerted an inhibitory effect on carotenoids accumulation and growth (<5% of control) at a concentration of 1µM, and at 48 h of cultivation as shown in Table 1 and Figure 1B. Once selected the appropriate concentration of each inhibitor, we proceeded to assess its effect on the synthesis of carotenoids, chlorophyll, β-carotene, protein and RNA in *D. salina* grown under carotenogenic and non-carotenogenic conditions. When *D. salina* cells were grown under conditions which enhances carotenoids accumulation and in presence of the highest concentration of fosmidomycin (200  μM, the highest response among the assayed concentrations), a decrease in the relative content in carotenoids was registered after four days of culture (Figure 2). This result represented, less than 75% of the content in the cells cultured under non-carotenogenic conditions (control). On the contrary, when *D. salina* cells were cultured under carotenogenic conditions and exposed to 1 μM mevinolin, this inhibitor did not exerted a significant change in the relative content of carotenoids during the exposure time (four days)  when compared to the cells cultured under non-carotenogenic conditions (control) (Figure 2).

**Figure 3:**
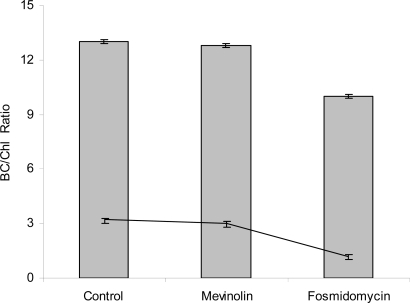
β-Carotene-to-chlorophyll ratio in *Dunaliella salina* exposed to metabolic inhibitors under carotenogenic conditions (Bars) and non-carotenogenic control (line). Values are means ± SD (each point represent n=3).

Analysing the ratio β-carotene to Chlorophyll ratio (Figure 3) during the exposure time to the inhibitors, highest ratios were registered under carotenogenic conditions exposed to mevinolin when compared to the non-carotenogenic cells exposed to fosmidomycin.

**Figure 4:**
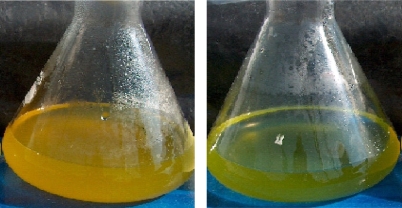
Culture flasks containing *D. salina* cells cultured under carotenogenic conditions and exposed to mevinolin (uninterrupted synthesis of β-carotene, left)  and to fosmidomycin (blocked synthesis of β-carotene, right) respectively.

Mevinolin treated *D. salina* cells continue to grow and synthesizing carotenoids, as observed in figure 4, cells turned orange-yellow and a decrease in cell density was observed and the β-carotene levels were similar to those of control cell cultures. Figure 4 shows green cells as indicative of photosynthetic growth and blocked synthesis of carotenoids and β-carotene in *D. salina* by fosmidomycin. The fact that the treatment with fosmidomycin did not completely suppress the system of renewal of its pools of isoprenoids and respective products from carotenogenesis in *Dunaliella salina*, viz, β-carotene indicates the effective inhibition of the essential regulatory step, DOXP reductoisomerase (generated under photosynthetic conditions) of the DOXP/MEP pathway    by fomidomycin which  is a structural analogue to 2-C-methylerythrose 4-phosphate, the intermediate in the enzymic reaction of DXR [20]. The results evidenced the absence of  HMGR, as well as  a suppression of the C5-units of biosynthesis, resulting in a shortage of the bulk isoprenoids like carotenoids, β-carotene, and chlorophylls (by phytol reduction, side chain of chlorophylls) at the level of chloroplast, the exclusive site of carotenogenesis in *D. salina*.

**Figure 5:**
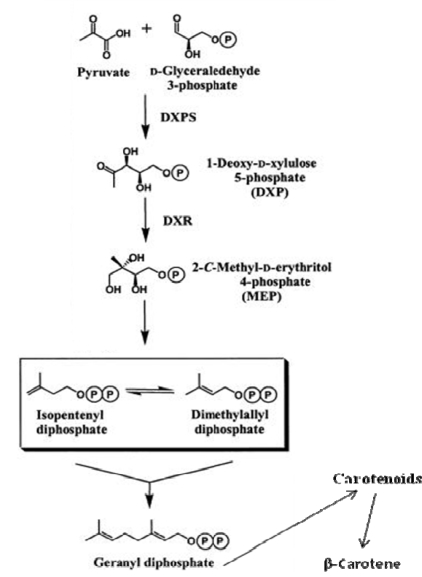
Supposed simplified pathway of the early steps of carotenogenesis and β-carotene accumulation in *Dunaliella salina* (modified after [21]).

All experiments were performed in order to favor growth and to try to enhance the incorporation of carbon (from carbon  dioxide) from air during photosynthesis. The photosynthetic autotrophic conditions promoted in cultures of Dunaliella leads to synthesis of pentose phosphate cycle substrates in the Calvin cycle from pyruvate pools, which conversion stimulate the apparent synthesis of the first precursors of the IPPP/DMAPP and lately incorporated into the chloroplast isoprenoids and carotenoids (Figure 5).

Carbon dioxide via photosynthesis has been reported as the main carbon source for isoprenoid biosynthesis via the mevalonate-independent methylerythritol 4-phosphate route in the marine diatoms *Phaeodactylum tricornutum* and *Nitzschia ovalis*  [22].

**Figure 6:**
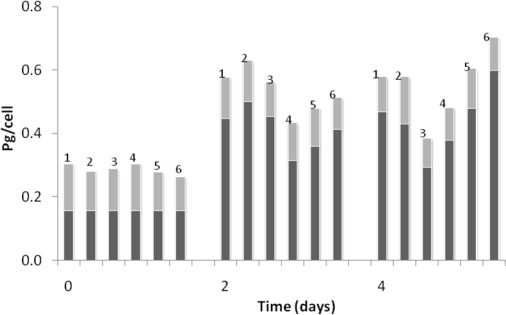
Protein (dark bar) and RNA content (gray bar)  in *D. salina*  during carotenogenesis and exposed to inhibitors: 1, Control (C); 2, C+ 1.0 μM mevinolin; 3-6=, C+ 50, 100, 150 and  200  μM fosmidomycin respectively.

The action of fomidomycin slightly decreased the content of protein in *D. salina*, under non-carotenogenic conditions (Figure 6). At 1.0 μM  mevinolin, there was no decrease in the content of protein. A similar trend was observed in RNA content during carotenogenesis. In principle, cellular RNA content was constant during carotenogenic conditions when *D. salina* was exposed to the assayed concentrations in mevinolin and fosmidomycin. Results in other Chlorophyte [23] have shown that total carotenoids were related to total proteins, suggesting that the enzyme (s) responsible for carotenoid synthesis must account for only a small part of total protein. This mechanism could indicate the importance of nitrogen to achieve adequate level of RNA and protein formation required for carotenoid biosynthesis during carotenogenesis.

Limited information exist on the important early steps of isorpenoid biosynthesis leading to carotenoid formation in *Dunaliella salina*. [10, 24] reported the biosynthesis of plastidic  isoprenoids in heterotrophic chlorophyte and non carotenogenic microalgae. *Dunaliella salina,* an obligate photoautotrophic microalga that cannot use dissolved organic compounds is one of the most important microalgae in biotechology considering its hyperproducing properties of β-carotene.

The role of carotenoid accumulation in *D. salina* dependent as it is on photosynthesis for its continued growth and survival, and their production requires redirection of a substantial amount of the cells' carbon resources into synthesis of isoprenoids. The accumulation of carotenoids in *D. salina* (β-carotene represents approx. 95% in dry weight) is considered  as a general response to stress conditions that presumably is of advantage to its survival in extreme natural conditions in which this alga grows [1].

Thus, the initiation of algal carotenogenesis exclusively occurs in the chloroplast as a result of the photoactivation of the carotenogenic enzymes [3]. We were unable to demonstrate a cytosolic branch of IPP biosynthesis. Our results suggest there is a low probability in *Dunaliella salina* cells of crosstalk between the chloroplast DXP/MEP and the cytosolic MVA pathways, as was previously reported in plants and certain algae [25, 20], since massive accumulation of carotenoids and β-carotene occurred only in the interthylakoid spaces of the chloroplast in this Chlorophyte.

The high difference in carotenoids accumulated in *D. salina* exposed to fosmidomycin under  carotenogenic and non-carotenogenic conditions can exclude the possible effect of the the stored isoprenoids and carotenoids during the short experimental period used here. Among the posible factors to be considered in the partial percentage of inhibition of carotenoids biosynthesis by fosmidomycin  (lower than 100 μM), we speculate and associate our result to a partial lack of uptake of fosmidomycin into cells by *D. salina* or inactivation of the action of this inhibitor throghout the culture period. In this work we have shown that isoprenoids-mediated carotenoids interference can be effectively applied by using carotenogenesis inducers, as well as inhibitors, viz,  fosmidomycin or mevinolin. Even when more significantly, the changes in pigments involved in photosynthesis (chlorophylls and carotenoids) are relatively bigger  than those in RNA and protein in *D. salina* isolated from the hipersaline lagoon  La   salina from Baja California Mexico.

## Figures and Tables

**Table 1:** Effect of the concentrations of mevinolin (Mev) and fosmidomycin (Fos) in growth, Chlorophyll and total carotenoids of *D. salina* under carotenogenic growth phase. Mean values indicate standard deviation (n = 3).

		Cellular growth (N × 10^6^ cells. ml^−1^) Days			
		1	2	7
Control (No Inhibitor)		1.1 ± 0.02	1.4 ± 0.01	1.6 ± 0.03
Mev (μM)	0.5	1.1 ± 0.01	1.2 ± 0.02	1.6 ± 0.01
	1	1.2 ± 0.01	1.3 ± 0.02	1.4 ± 0.02
	2	1.1 ± 0.04	1.3 ± 0.01	1.5 ± 0.02
Fos (μM)	50	1.1 ± 0.02	1.1 ± 0.01	0.5 ± 0.03
	100	1.2 ± 0.02	1.0 ± 0.03	0.4 ± 0.01
	150	1.1 ± 0.03	1.0 ± 0.02	0.4 ± 0.02
	200	0.8 ± 0.01	0.8 ± 0.02	0.3 ± 0.01
Chlorophyll (pg. cell^−1^)				
Control (No Inhibitor)		0.4 ± 0.01	0.5 ± 0.02	0.6 ± 0.02
Mev (μM)	0.5	0.4 ± 0.02	0.4 ± 0.03	0.5 ± 0.02
	1	0.4 ± 0.02	0.5 ± 0.01	0.5 ± 0.02
	2	0.5 ± 0.02	0.5 ± 0.01	0.5 ± 0.03
Fos (μM)	50	0.4 ± 0.01	0.4 ± 0.02	0.3 ± 0.01
	100	0.4 ± 0.02	0.4 ± 0.01	0.1 ± 0.02
	150	0.3 ± 0.02	0.3 ± 0.01	0.1 ± 0.01
	200	0.3 ± 0.01	0.3 ± 0.02	0.1 ± 0.01
Total carotene (pg. cell^−1^)				
Control (No Inhibitor)		1.2 ± 0.03	1.5 ± 0.02	1.7 ± 0.01
Mev (μM)	0.5	1.3 ± 0.02	1.4 ± 0.01	1.7 ± 0.02
	1	1.3 ± 0.04	1.5 ± 0.02	1.8 ± 0.01
	2	1.5 ± 0.02	1.6 ± 0.03	1.8 ± 0.02
Fos (μM)	50	1.1 ± 0.01	1.1 ± 0.03	0.8 ± 0.01
	100	0.9 ± 0.02	0.9 ± 0.02	0.4 ± 0.01
	150	0.7 ± 0.02	0.6 ± 0.01	0.3 ± 0.03
	200	0.4 ± 0.01	0.4 ± 0.01	0.2 ± 0.01
